# [^18^F]DPA-714: Direct Comparison with [^11^C]PK11195 in a Model of Cerebral Ischemia in Rats

**DOI:** 10.1371/journal.pone.0056441

**Published:** 2013-02-13

**Authors:** Hervé Boutin, Christian Prenant, Renaud Maroy, James Galea, Andrew D. Greenhalgh, Alison Smigova, Christopher Cawthorne, Peter Julyan, Shane M. Wilkinson, Samuel D. Banister, Gavin Brown, Karl Herholz, Michael Kassiou, Nancy J. Rothwell

**Affiliations:** 1 Faculty of Medical and Human Sciences, University of Manchester, Manchester, United Kingdom; 2 Wolfson Molecular Imaging Centre, University of Manchester, Manchester, United Kingdom; 3 Service Hospitalier Frédéric Joliot – Commissariat à l'Energie Atomique, Orsay, France; 4 Faculty of Life Sciences, University of Manchester, Manchester, United Kingdom; 5 Brain Injury Research Group, Manchester Academic Health Sciences Centre, Salford Royal NHS Foundation Trust, Salford, United Kingdom; 6 North Western Medical Physics, Christie Hospital, Manchester, United Kingdom; 7 School of Chemistry, University of Sydney, Sydney, Australia; 8 Discipline of Medical Radiation Sciences, University of Sydney, Sydney, Australia; 9 Brain and Mind Research Institute, University of Sydney, Sydney, Australia; The University of Chicago, United States of America

## Abstract

**Purpose:**

Neuroinflammation is involved in several brain disorders and can be monitored through expression of the translocator protein 18 kDa (TSPO) on activated microglia. In recent years, several new PET radioligands for TSPO have been evaluated in disease models. [^18^F]DPA-714 is a TSPO radiotracer with great promise; however results vary between different experimental models of neuroinflammation. To further examine the potential of [^18^F]DPA-714, it was compared directly to [^11^C]PK11195 in experimental cerebral ischaemia in rats.

**Methods:**

Under anaesthesia, the middle cerebral artery of adult rats was occluded for 60 min using the filament model. Rats were allowed recovery for 5 to 7 days before one hour dynamic PET scans with [^11^C]PK11195 and/or [^18^F]DPA-714 under anaesthesia.

**Results:**

Uptake of [^11^C]PK11195 *vs* [^18^F]DPA-714 in the ischemic lesion was similar (core/contralateral ratio: 2.84±0.67 *vs* 2.28±0.34 respectively), but severity of the brain ischemia and hence ligand uptake in the lesion appeared to vary greatly between animals scanned with [^11^C]PK11195 or with [^18^F]DPA-714. To solve this issue of inter-individual variability, we performed a direct comparison of [^11^C]PK11195 and [^18^F]DPA-714 by scanning the same animals sequentially with both tracers within 24 h. In this direct comparison, the core/contralateral ratio (3.35±1.21 *vs* 4.66±2.50 for [^11^C]PK11195 *vs* [^18^F]DPA-714 respectively) showed a significantly better signal-to-noise ratio (1.6 (1.3–1.9, 95%CI) fold by linear regression) for [^18^F]DPA-714.

**Conclusions:**

In a clinically relevant model of neuroinflammation, uptake for both radiotracers appeared to be similar at first, but a high variability was observed in our model. Therefore, to truly compare tracers in such models, we performed scans with both tracers in the same animals. By doing so, our result demonstrated that [^18^F]DPA-714 displayed a higher signal-to-noise ratio than [^11^C]PK11195. Our results suggest that, with the longer half-life of [^18^F] which facilitates distribution of the tracer across PET centre, [^18^F]DPA-714 is a good alternative for TSPO imaging.

## Introduction

For more than a decade an increasing body of evidence has demonstrated that neuroinflammation is involved in many acute or chronic neurological conditions and can be used as a biomarker of disease progression [1]–[6]. In 1977, binding studies characterised in peripheral organs a new benzodiazepine binding sites, the peripheral benzodiazepine receptor (PBR), and specific ligands such as PK11195 or Ro5-4864 [7]–[9] for this binding site were identified. It has since been renamed translocator protein 18 kDa TSPO [10]. [^11^C] and [^123^I] labelled PK11195 were initially developed to follow brain lesions using PET or SPECT imaging [11]–[15]. However, its pharmacological properties (high non-specific binding, poor signal to noise ratio) have limited to some extent its ability to detect subtle changes in TSPO expression and extensive work has been required to improve quantification and modelling of [^11^C]PK11195 [16]–[18]. This has led to extensive efforts to develop new radiotracers more suitable for TSPO imaging. Several promising candidates have been evaluated pre-clinically, and for some of them clinical evaluation has started (for review see [19], [20]). However, as reviewed recently by Luus *et al.* [21], it appears that thorough validation of each candidate is necessary before clinical imaging can commence; an example of this is the existence of patients expressing low or high affinity TSPO binding sites (or a combination of both, i.e. “mixed binders”) for [^11^C]PBR28 [22], [23].

Among the TSPO radiotracers developed in the recent years, [^18^F]DPA-714 has shown great promise. [^18^F]DPA-714 has been evaluated in a model of acute neuroinflammation induced by stereotaxic injection of alpha-amino-3-hydroxy-5-methyl-4-isoxazolepropionic acid (AMPA), in which it showed enhanced imaging characteristics compared to [^11^C]PK11195 [24]. However, AMPA induces acute neuroinflammation, and although very useful for first assessment of new TSPO tracers, it has no clinical relevance. Since then [^18^F]DPA-714 has been used to follow the time-course of TSPO expression following experimental stroke in rats, and yielded results similar to those described previously with PK11195 [25], [26]. However, this latter study did not compare [^18^F]DPA-714 with [^11^C]PK11195 directly. More recently, Doorduin *et al.* [27] compared [^18^F]DPA-714 and [^11^C]PK11195 in a model of Herpes encephalitis (HSE), and showed no significant differences between the uptake of these two tracers in all regions of interest (ROI) studied, however tracers were not compared in the same animals and variability of neuroinflammation was very high (variability: 45±11% for [^11^C]PK11195 and 39±20% for [^18^F]DPA-714). Therefore, further assessment directly comparing [^18^F]DPA-714 and [^11^C]PK11195 is needed.

TSPO expression has been detected robustly after stroke in experimental animal models and patients [25], [26], [28]–[32], so we compared [^11^C]PK11195 and [^18^F]DPA-714 after focal cerebral ischaemia in rats using *in vivo* PET imaging. This experimental approach is particularly relevant to further evaluate the potential translational use of [^18^F]DPA-714 in a clinical setting.

## Methods

### Induction of focal cerebral ischaemia in rats

Studies were conducted on male Sprague-Dawley rats (n = 15) (Charles River, Margate, Kent, UK) in a first set of experiments. As our stroke model became unreliable in term of infarct volume in Sprague-Dawley rats, we conducted a study comparing Sprague-Dawley rats and Wistar rats looking only at infarct size by histology; and in our hands Wistar rats provided more reliable ischemic brain damages than Sprague-Dawley (data not shown). Based on these findings and to complete the dual scans PET study (sequential scans [^11^C]PK11195 - [^18^F]DPA-714) with a higher success rate of animals presenting a stroke, five Wistar rats (Charles River, Margate, Kent, UK) were scanned with [^11^C]PK11195 and [^18^F]DPA-714 sequentially. The PET data from these five animals were pooled with the results of the four Sprague-Dawley rats scanned with both [^11^C]PK11195 and [^18^F]DPA-714 of the first set of experiments. Distribution of all the animals across the groups can be found in Table 1.

**Table 1:** Distribution of the animals in the experimental groups.

	Tracer used		
PET Scanner	[^11^C]PK11195 only	[^18^F]DPA-714 only	Dual scans‡
HIDAC*	7	4	4 (−2)†
Inveon#	-	-	5
TOTAL (*n*)	7	4	9 (7)

* Sprague-Dawley (SD) rats only.

† In bracket, two SD rats showed no significant infarct by immunohistochemistry examination.

# Wistar rats only.

‡ Rats were scanned sequentially with [^11^C]PK11195 and [^18^F]DPA-714; six rats were scanned first with [^11^C]PK11195 then [^18^F]DPA-714 and three rats were scanned first with [^18^F]DPA-714 then [^11^C]PK11195.

Animals weighted 300 to 400 g. The animals were kept under a 12 h light–dark cycle with free access to food and water. All procedures were carried out in accordance with the Animals (Scientific Procedures) Act 1986, the specific project licence was approved by the UK Home Office.

Focal cerebral ischaemia was induced by 60 min transient occlusion of the middle cerebral artery (MCAO) under isoflurane anaesthesia (induction 4% and maintained 1.5% in 70% N_2_O and 30% O_2_ mixture) as described by Longa *et al.* [33]. Core body temperature was maintained throughout the procedure at 37.0±0.5°C by a heating blanket (Homeothermic Blanket Control Unit; Harvard Apparatus Limited). MCAO was verified by a >60% drop in cerebral blood flow (CBF) monitored by laser Doppler (Moor Instruments Ltd, Devon, UK). After 60 min, the filament was withdrawn to restore CBF.

### Positron Emission Tomography scans and data acquisition

Five to seven days after MCAO, rats were anaesthetised by isoflurane inhalation (induction: 5% and thereafter 2–2.5%) in oxygen. In total, 7 animals were scanned with [^11^C]PK11195 and 4 with [^18^F]DPA-714 only. Nine rats were scanned sequentially with both [^11^C]PK11195 and [^18^F]DPA-714 within 24 h (6 rats were scanned with [^11^C]PK11195 first and then with [^18^F]DPA-714 (3 h later or more) and 3 rats were scanned with [^18^F]DPA-714 first and with [^11^C]PK11195 the following day). As control, three naive rats (no MCAO) were also scanned.

[^11^C]PK11195 and [^18^F]DPA-714 were synthesized as described elsewhere [11], [34], [35] (specific activity at injection time: [^11^C]PK11195: 15.35–258.99GBq/µmol; [^18^F]DPA-714: 14.93–156.43 GBq/µmol), and injected intravenously in the tail vein as a bolus ([^11^C]PK11195: 11.7–31.5 MBq [0.05–0.89 nmol]; [^18^F]DPA-714: 15.0–24.5 MBq [0.09–1.42 nmol] for the HIDAC scans, and [^11^C]PK11195: 22.2–35.5 MBq [0.12–0.33 nmol]; [^18^F]DPA-714: 22.2–35.5 MBq [0.35–1.20 nmol] for the Inveon scans).

In a first set of experiments, whole-body images were acquired for 1 h in list-mode with a non-rotating 16-module quad-HIDAC PET camera (Oxford Positron Systems, Weston-on-the-Green, UK) with a resolution of 1 mm^3^ (Spatial resolution, FWHM) [36], [37]. The list-mode data were reconstructed directly into 5 min time-frames images (without resorting to histogramming) via the one-pass-list-mode-expectation maximisation (OPL-EM) algorithm [38] with one iteration of 16 sub-sets into images of dimensions 120^2^ (transaxially)×240 (axially) with isotropic 1 mm^3^ voxels. Absolute calibration of the images was achieved by reference to a [^22^Na] source imaged in the field of view in each scan. This had been validated with a uniformly filled mouse-sized [^18^F] phantom imaged over two hours. Dynamic images were calibrated in kBq.cm^−3^.

The complementary dual scans experiments were performed on a Siemens Inveon® PET-CT scanner as the HIDAC scanner had been replaced. The acquisition protocol consisted of the following parameters: a CT scan was performed prior the PET acquisition to obtain the attenuation correction factors, the time coincidence window was set to 3.432 ns and the levels of energy discrimination were set to 350 keV and 650 keV. The list mode acquisition data files were histogrammed into 3D sinograms with a maximum ring difference of 79 and span 3. The list mode data of the emission scans were sorted into 16 dynamic frames. Finally, the emission sinograms (each frame) were normalized, corrected for attenuation, scattering and radioactivity decay, and reconstructed using OSEM3D (16 subsets and 4 iterations) into images of dimensions 128^2^ (transaxially)×159 (axially) with 0.776×0.776×0.796 mm voxels.

Respiration and temperature were monitored throughout using a pressure sensitive pad and rectal probe (HIDAC: Model 1025L interface and PC-SAM software, SA Instruments, NJ USA; Inveon: BioVet, m2m imaging crop, USA). Body temperature was maintained by use of a heating and fan module controlled by the rectal probe via the interface controlled by the PC-SAM (HIDAC) or BioVet (Inveon) software.

At the end of the last PET scan, rats were quickly decapitated and the brains were quickly removed and immediately frozen in isopentane in dry ice. The brains were stored at −80°C until cut with cryomicrotome in adjacent 20 µm thick coronal sections. Brain sections were then stored at −80°C until used for autoradiography or immunohistochemistry.

### Image analysis

Images were segmented using the Local Means Analysis (LMA) method and the organ mean Time Activity Curves were corrected for Partial Volume Effect. The correction method combined the Geometric Transfer Matrix (GTM) method and the ROIopt method both described by Maroy *et al.* [39], [40] and previously used [37]. Both methods were applied using the BrainVisa/Anatomist framework (http://brainvisa.info/). PET images were co-registered with the rat MRI template and its associated intracranial mask and published by Schwarz et al. [41] and generously provided by GSK. Segmented ROI were selected within the volume of the brain mask provided with the MRI template, the cerebellum was delineated based on the co-registration with the MRI template. For rats scanned with both [^11^C]PK11195 and [^18^F]DPA-714, quantification of anatomical structures irrespectively of infarct localisation was achieved by use of the ROIs from the 3D brain atlas co-registered with the MRI template.

Automatic segmentation of the volume within the MRI intracranial mask had the advantage of delineating user-independent ROIs. For both tracers the following five ROIs were automatically segmented and labelled as: (1) core (ROI covering the infarct in the MCAO territory and/or with the highest uptake), (2) edge-1 (ROI around the core ROI and/or with the second highest uptake), (3) edge-2 (ROI around the core ROI and/or with the 3^rd^ highest uptake), (4) contralateral ROI (ROI with the lowest uptake) and (5) skull edges (5^th^ ROI segmented in some animals, located on the edge of the MRI template, this ROI was not included in the analysis). In control animal, a single ROI covering the whole brain was segmented (data not shown).

### Immunohistochemistry

For all the rats used for the PET study, astrogliosis, microglial activation and neuronal loss were checked by immunohistochemistry staining for GFAP, CD11b and MAP2 respectively. Blood brain barrier (BBB) integrity was checked by immunohistochemistry against Claudin-5 to visualise tight-junction and IgG to visualise potential large protein diffusion through a damaged BBB.

For all the procedure described below Phosphate Buffered saline (PBS) at 100 mM was used. Frozen rat brain sections were post-fixed in paraformaldehyde (4% in PBS) for 30 min and washed (6×5 min) in PBS. Sections were permeabilized with 30 min of incubation in 0.1% Triton X-100 containing 2% normal donkey serum in PBS to block non-specific binding. Without further washing, sections were incubated overnight at 4°C with primary antibodies in 2% normal donkey serum/0.1% Triton X-100 in PBS. Triple immunohistochemistry staining was performed against glial fibrillary acidic protein (GFAP) with rabbit anti-cow GFAP (Dako, 1∶1000); CD11b (Ox42) with mouse anti-rat CD11b (Serotec, 1∶1000) and MAP2 with chicken anti-mouse MAP2 (Abcam 1∶2000). Adjacent sets of sections were incubated with rabbit anti-rat Claudin-5 Rabbit (Abcam 1∶500). Sections were then washed (3×10 min) in PBS and incubated for 2 h at room temperature with secondary antibodies (AlexaFluor 488 nm donkey anti-mouse IgG, AlexaFluor 594 nm donkey anti-rabbit IgG (Molecular Probes, Invitrogen) and AMCA-conjugated 350 nm donkey anti-chicken IgG (Jackson ImmunoResearch laboratories, inc.), all 1∶500 in 2% normal donkey serum/0.1% Triton X-100 in PBS) and then washed again (3×10 min) in PBS. BBB assessment sections were incubated with LICOR® secondary antibodies IRDye® 800CW Donkey anti-rabbit and IRDye® 680LT Goat anti-Rat IgG. Sections were mounted with a Prolong Antifade kit (Molecular Probes, Invitrogen); those incubated without the primary antibodies served as negative controls.

Images were collected on a Olympus BX51 upright microscope using a 4×/0.13, 10×/0.30 or 40×/0.50 UPlanFLN objectives and captured using a Coolsnap ES camera (Photometrics) through MetaVue Software (Molecular Devices). Specific band pass filter sets were used to prevent bleed through from one channel to the next. Images were then processed and analysed using ImageJ (http://rsb.info.nih.gov/ij).

Claudin-5 and IgG images were acquired using a LICOR Odyssey® imager system, set-up at its highest resolution (21 µm) for individual section.

### Autoradiography

[^18^F]DPA-714 (92.4 GBq/µmol; 18 nM) autoradiography was performed using 20 µm brain sections from five Sprague-Dawley rats presenting an average ischaemic lesion on the immunohistochemistry staining. Using adjacent sections, we assessed specific binding for TSPO by adding an excess of unlabeled PK11195 or unlabelled DPA-714 (20 µM). Sections were incubated for 60 min in Tris buffer (Trizma preset crystals (Sigma, UK) adjusted at pH 7.4 at 4°C, 50 mM, with 120 mM NaCl) and then were rinsed twice for 2 min with cold buffer, followed by a quick wash in cold distilled water and dried before exposition onto Phosphor-Imager screen overnight. Autoradiographs were visualized and analyzed using AIDA software (Raytest GmbH, Germany). Regions of interest (ROI) were manually drawn on the lesion, the surrounding tissue in the ipsilateral and the contralateral hemisphere. Binding in the ROIs is expressed as intensity of the photostimulated luminescence (PSL) per pixel.

### Statistical analysis

For each tracer, comparison between ROIs binding values from the autoradiographic study was performed using ANOVA and Scheffe's multiple comparison tests.

Non-parametric Mann-Whitney test was used to compare [^11^C]PK11195 and [^18^F]DPA-714 uptake values when different animals were used. In animals scanned with both radiotracers, Wilcoxon test was used to compare [^11^C]PK11195 *vs* [^18^F]DPA-714 uptake values for each ROI and to compare between ROIs for each tracer (Statview 5.0.1 software, SAS Institute Inc., Cary, NC, USA). All data are expressed as mean±SD.

Regression analysis of the dual scan data was performed with GraphPad Prism version 5.04 for Windows (GraphPad Software, San Diego California USA, www.graphpad.com), best fit was chosen by comparing linear, power (*y-a.x^b^*), second order polynomial and exponential fits using extra sum-of-square *F* test and Akaike information criterion for relative goodness of fit, tests were done with and without constraints (i.e. fitted or fixed *y* intercept for x = 0).

## Results

### *Ex vivo* analysis

**Figure 1:**
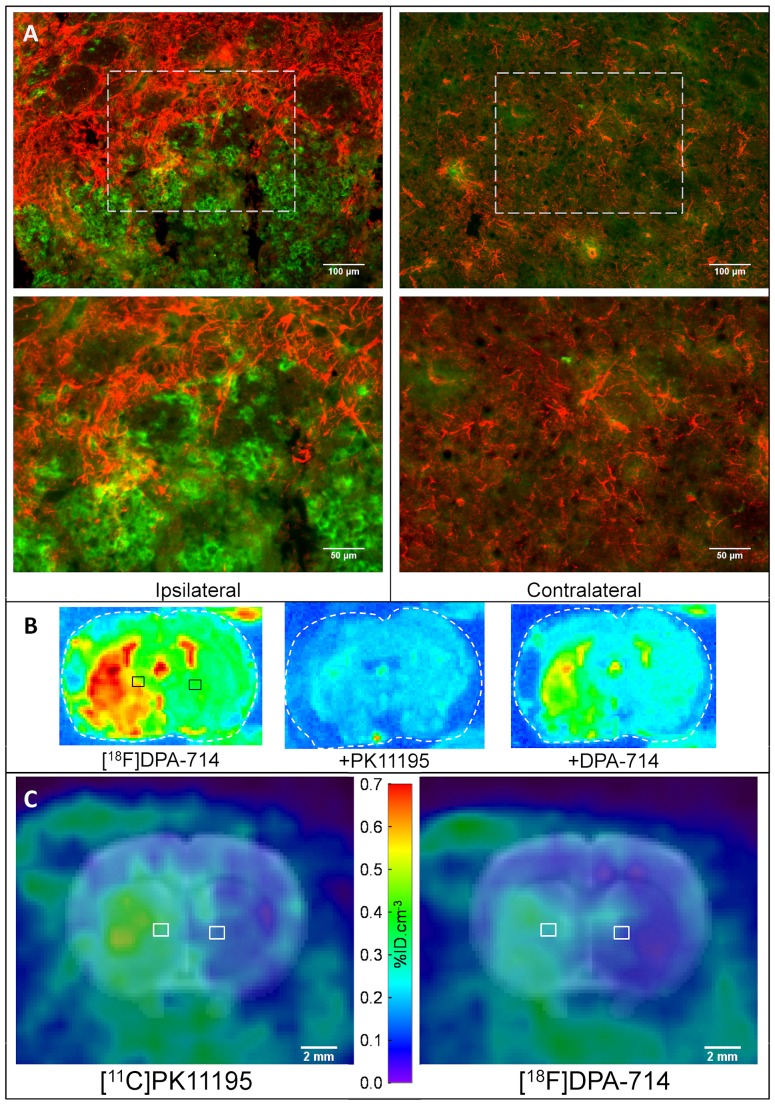
Immunohistochemistry (A), autoradiography (B), and PET images (C) from the same animal post-MCAO. The black squares on the autoradiographs (**B**) represent the approximate localisation of the immunohistochemistry (**A**) on adjacent brain sections from the same animal. Characteristically, astrocytes (GFAP antibody, red) delineated the edge of the infarct and abundant microglial cells (CD11b antibody, green) were found in the core of the lesion (left panel), whereas little or no activated astrocyte or microglia could be observed in the contralateral side (right panel). (**B**) Representative images of autoradiography performed on rat coronal brain sections incubated with [^18^F]DPA-714 (18 nM) alone or in presence of PK11195 or unlabelled DPA-714 (20 µM). (**C**) [^11^C]PK11195 and [^18^F]DPA-74 PET sum images (20–60 min) co-registered with the MRI template of the same animal at similar coronal level.

The presence of a large number of activated microglial cells, as shown by CD11b immunohistochemistry (Figure 1A) confirmed the presence of an ischaemic lesion and correlated well with the presence of increased [^18^F]DPA-714 or [^11^C]PK11195 uptake when compared to normal or contralateral brain tissue (Figure 1A and 1B). Moreover, such lesions were characteristically surrounded by a scar of reactive astrogliosis (Figure 1A). Similarly, TSPO expression, as visualised by autoradiography, correlated well with the localisation seen on the PET images (Figure 1B, 1C). Quantification of the autoradiography revealed that the specific binding of [^18^F]DPA-714 was significantly higher (3.6 fold) in the lesion than in the contralateral side. Overall, the immunohistochemistry confirmed post-mortem that all animals single-scanned with [^11^C]PK11195 (n = 7) had a large infarct (i.e. including striatal and cortical areas). From 4 animals single-scanned with [^18^F]DPA-714, three had a large infarct and one had a small infarct limited to the striatum. From the nine animals scanned successively with [^11^C]PK11195 and [^18^F]DPA-714, four had a large infarct with cortical damages, three had a small infarct limited to the striatum and two had diffuse and very limited microglial activation and astrogliosis in the MCA territory indicating very little ischaemic damages, despite a significant decrease (≥60% for 60 min) in CBF during MCAO.

The regions in which tight-junctions were missing or had not been fully restored (as shown by a weak Claudin-5 immunostaining) mostly overlap the areas where diffuse IgG staining was visible in the brain parenchyma rather than in blood vessels as observed in the contralateral tissue (Figure S1). This analysis revealed that, in all animals, the extent of BBB damages were proportional to the brain damage as observed by CD11b and GFAP immunostaining at five to seven days post-MCAO.

### *In vivo* PET imaging

**Figure 2:**
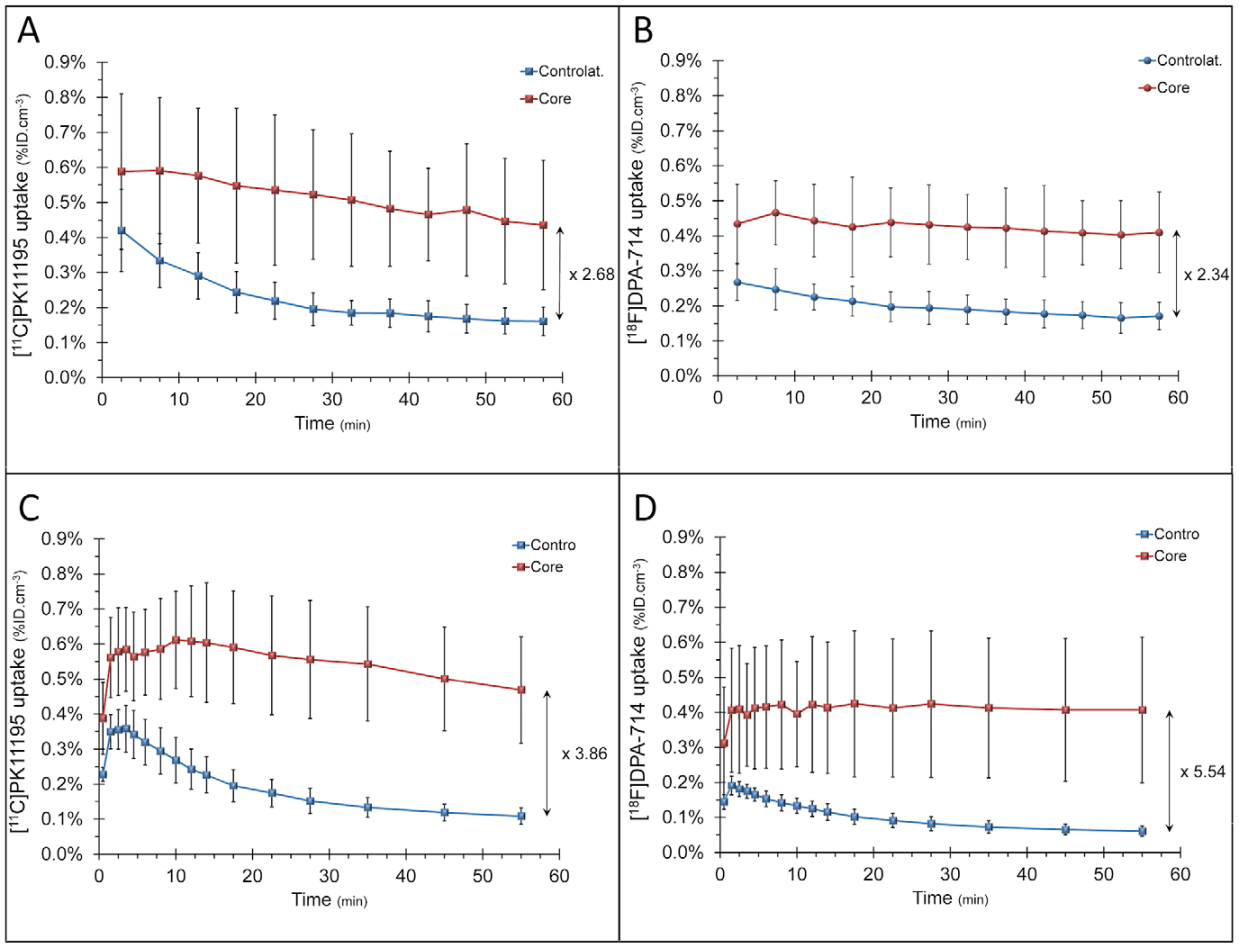
Time-activity curves of [^11^C]PK11195 (A and C) and [^18^F]DPA-714 (B and D) in the core of the lesion and the contralateral area (animals with visible infarct only) (A, B: HIDAC PET scanner, single tracer and dual scans pooled; C, D: Inveon PET-CT scanner).

[^11^C]PK11195 and [^18^F]DPA-714 had slightly different pharmacokinetic profile (Figure 2A & C *vs* 2B & D ). However, the pharmacokinetic of each tracer was similar when comparing between scanners (HIDAC *vs* Inveon) (Figures 2A *vs* 2C and 2B *vs* 2D). The edge-1 and edge-2 ROIs were located around the core of the infarct, likely to correspond to the astrocytic scar seen on the GFAP immunostaining (Figure 1A).

No significant differences in uptake between the two tracers when comparing [^11^C]PK11195 and [^18^F]DPA-714 groups of animals scanned with only one of the tracer or when comparing groups in which all the animals with visible infarct (single and dual scans) were pooled together. The [^18^F]DPA-714 uptake values in the segmented contralateral ROI were not significantly different from the uptake values in control rats (0.133±0.064%ID.cm^−3^ *vs* 0.174±0.014%ID.cm^−3^), suggesting an accurate delineation of the ROI of reference. The 20–60 min average uptake values in the contralateral, cerebellum and edge-2 ROIs obtained with the Inveon were significantly lower than those obtained with the HIDAC (data not shown). Uptake in high uptake ROIs (core, edge-1) were not different between scanners.

**Figure 3:**
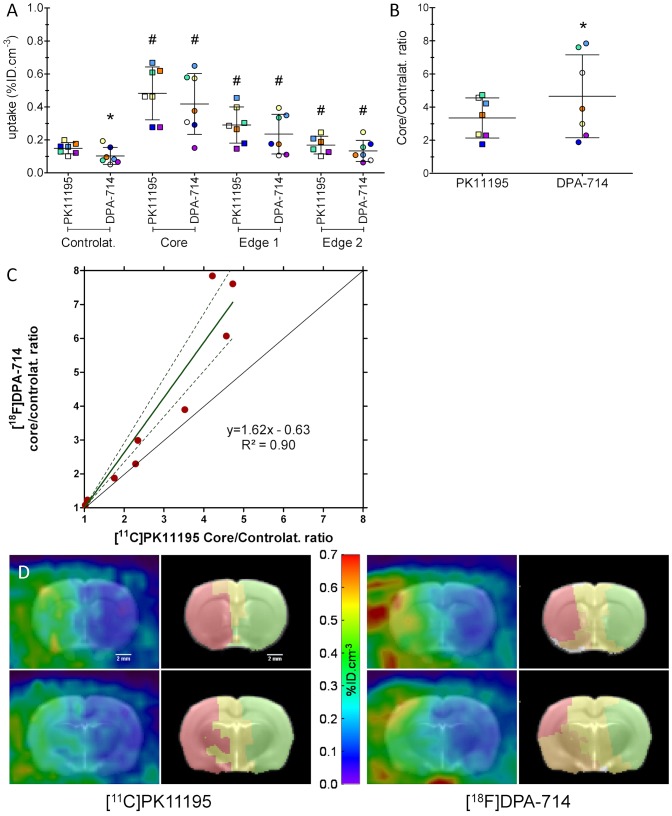
[^11^C]PK11195 and [^18^F]DPA-714 mean uptake values between 20 and 60 min post-injection (expressed as percentage of injected dose per cm^3^, mean±SD (A) and Core/contralateral ROIs ratio (B)) of the 7 animals scanned with both tracers successively within 24 h (2 animals with no lesion are not shown on these graphs, although there exclusion did not affect the outcome of the statistical analysis). * Significantly different from [^11^C]PK11195 values, # significantly different from the contralateral side of the same tracer (Wilcoxon paired test, p<0.05). (**C**) Correlation between [^11^C]PK11195 and [^18^F]DPA-714 core/contralateral ROIs ratio (all animals included) (dotted line = 95% confidence interval). (**D**) On the left of each panel, PET images shown are summed images between 20 and 60 min after injection of [^11^C]PK11195 (left panel) and [^18^F]DPA-714 (right panel) co-registered with the MRI template. The ROIs automatically segmented from the corresponding PET images are shown on the right part of each panel. The ROIs are: infarct core (red; ROI covering the infarct in the MCAO territory and/or with the highest uptake), edge-1 (orange; ROI around the MCAO territory and/or with the 2^nd^ highest uptake), edge-2 (yellow; ROI around the MCAO territory and/or with the 3^rd^ highest uptake), contralateral ROI (green; ROI with the lowest uptake) and skull edges (white; 5^th^ ROI segmented in some animals, located on the edge of the skull, this ROI was not included in the statistical analysis).

To limit the impact of the inter-individual variability of the stroke model, nine rats were scanned with both [^11^C]PK11195 and [^18^F]DPA-714 within 24 h, allowing a true direct comparison of the two tracers; two of these rats did not have a significant infarct at post-mortem examination. Uptake values for [^11^C]PK11195 and [^18^F]DPA-714 in the core, edge-1 and edge-2 ROIs were significantly higher than the uptake value measured in their respective contralateral ROI (Figure 3A). [^18^F]DPA-714 uptake in the core ROI was not significantly different from [^11^C]PK11195 uptake (Figure 3A). However, the [^18^F]DPA-714 uptake in the contralateral ROI was significantly lower than [^11^C]PK11195 uptake (Figure 3A), leading to a significantly higher core/contralateral ratio for [^18^F]DPA-714 (Figure 3B). [^18^F]DPA-714 core/contralateral ratio was significantly correlated to [^11^C]PK11195 core/contralateral ratio (R^2^ = 0.90), demonstrating a ∼1.6 (1.3∼1.9 95% CI) fold improvement for [^18^F]DPA-714 over [^11^C]PK11195 (Figure 3C).

We also performed data modelling using the SRTM [18], but found that both ligands had tracer delivery ratio values (R_1_ = k_1_/k′_1_) higher than 1 (Table S1), which indicated a faster tracer delivery in the infarct. To account for this potential artefact, we also modelled the data setting R_1_ = 1. In both conditions, binding potential (BP_ND_) values were significantly higher for [^18^F]DPA-714 than for [^11^C]PK11195 in the core of the lesion. However, values of R_1_>1, taken together with the disrupted BBB as shown by immunostaining, indicated that the conditions of the SRTM were not fulfilled and suggested that the interpretation of the binding potential should be done with caution.

To check whether the size and shape of the ROIs might have influenced the quantification and comparison, we also quantified the images using a simplified brain atlas co-registered with the MRI template (Figure S2), therefore identical ROIs were applied to both the [^11^C]PK11195 and [^18^F]DPA-714 images. This approach gave similar results to the automated segmentation in ischaemic brain structures (such as the caudate-putamen).

## Discussion

In order to test further the suitability of [^18^F]DPA-714 as a replacement for [^11^C]PK11195, the present study compared both tracers following focal brain ischaemia, which is a more clinically relevant model than excitotoxic lesion. Importantly, previous studies comparing [^11^C]PK11195 and DPA-174 [24], [27] did not perform sequential scans with both tracers as performed here to compare uptake of each tracer in the same animals. We are reporting here results showing that *i)* to truly compare tracers, sequential scans with both tracers, in our case [^18^F]DPA *vs* [^11^C]PK11195 is crucial and *ii)* [^18^F]DPA-714 has a great potential as a TSPO tracer to replace [^11^C]PK11195.

Due to the necessity for TSPO imaging over several days [26], [28], animal welfare considerations made it necessary to use a short (60 min) MCAO duration, which is known to produce lesions more variable in size [42], [43]. In terms of uptake values, the [^18^F]DPA-714 uptake reported here was comparable with published data following MCAO [25] in both the core of the infarct (0.42±0.15%ID.cm^−3^ *vs* ∼0.6±0.1%ID.cm^−3^) and the contralateral ROI (0.13±0.06%ID.cm^−3^ *vs* ∼0.15%ID.cm^−3^, present study *vs* Martin *et al.* [25]).

In our first set of single scanned animals, the high variability in the severity of the ischaemic lesion led to high variability in induced neuroinflammation as seen by both immunohistochemistry and PET imaging. This clearly hampered the comparison of the two radiotracers in different groups of animals. Indeed, when animals were scanned only with [^11^C]PK11195 or [^18^F]DPA-714, the overall absence of differences between the 2 tracers contrasted with previous data using AMPA-induced striatal lesions, in which [^18^F]DPA-714 uptake was 1.89 fold better than [^11^C]PK11195 uptake (ipsi/contralateral uptake ratio: [^18^F]DPA-714: 4.30±0.30 *vs* [^11^C]PK11195 2.27±0.08) [24]).

Therefore, one way of avoiding the problem was to scan the same animals with both [^11^C]PK11195 and [^18^F]DPA-714 successively at 5 and 6 or 6 and 7 days post-MCAO, as TSPO has been shown to change only slightly over 24 h [25], [26], [28]. However, this also introduces operational challenges such as the requirements of serial radiosynthesis within 24 h for a single experiment. When comparing [^11^C]PK11195 and [^18^F]DPA-714 uptake values from animals that were scanned with both tracers successively, we could demonstrate that the [^18^F]DPA-714 uptake in the contralateral (healthy) tissue was significantly lower than the [^11^C]PK11195 uptake, leading to a significantly better contrast between the core of the lesion and the contralateral tissue with values similar to those reported by Chauveau *et al*.[24]. We also show that [^18^F]DPA-714 uptake is linearly correlated to [^11^C]PK11195 uptake giving an advantage in term of core/contralateral ratio to DPA-714 over PK11195 by a factor 1.3∼1.9 (95% CI). If the correlation plot suggests that for modest increases in TSPO expression the difference between the two radiotracers will likely be small; the fact that the difference between the two tracer comes from a significantly lower uptake in the contralateral ROI supports the hypothesis that [^18^F]DPA-714 is likely to be a more sensitive tracer than [^11^C]PK11195 because of a lower non-specific signal or binding. These results were also confirmed by the modelling data (Table S1). However, we did observe R_1_ fitted values above 1 for both tracers suggesting a faster delivery in the core of the lesion than in the reference ROI. This clearly indicates that *i)* BBB status must always be checked if the animal model or the clinical neuropathological conditions is likely to disrupt the BBB and *ii)* that, although very challenging in small animals, arterial blood input function needs to be assessed whenever possible.

These findings clearly indicate that the impact of the models chosen to screen new tracers is of importance. Despite a weak relevance to clinical observation, excitotoxic lesions can be used as a rapid way of testing a tracer (i.e. brain penetration of the tracer, signal to noise ratio, specificity) as these models produce very consistent lesion and are easy and quick to set-up and perform. However, more clinically relevant models, such as stroke or encephalitis, must be used to check the robustness of the tracer in various conditions that may exhibit more variable level of neuroinflammation. This is well illustrated by the work of Doorduin *et al.* showing no differences in specific binding between [^18^F]DPA-714 and [^11^C]PK11195 in a model of Herpes encephalitis (HSE), although [^11^C]DPA-713 specific binding was significantly higher in 6 of the 11 ROIs studied [27]. The difficulty in comparing tracers in HSE [27] or MCAO models arises from the potential high inter-individual variability of the neuroinflammation induced (mean variability (%) of the specific binding measure in HSE model across ROIs: [^11^C]PK11195: 45±11%, [^11^C]DPA-713: 65±16% and [^18^F]DPA-714: 39±20%, from Table 5, Doorduin *et al.* [27]; mean variability (%) of the uptake values in MCAO model across ROIs in the present study: [^11^C]PK11195: 29±9%, [^18^F]DPA-714: 32±12%) when compared with more robust models such as excitotoxic lesion (mean variability (%) of the uptake values in AMPA model across ROIs: [^11^C]PK11195: 11±3%, [^11^C]DPA-713: 15±4% [44] and [^18^F]DPA-714: 7±2%); and the fact that independent animals injected with either [^11^C]PK11195 or alternative tracers such as [^18^F]DPA-714 were compared. However, it is noteworthy that such models are likely to be closer to the high variability observed amongst patients in a clinical situation.

We used an automatic segmentation method to define ROIs to avoid the bias inherent in manual methods. The automatic segmentation produced different ROIs for [^18^F]DPA-714 and [^11^C]PK11195 matching the highest, medium and lowest level (respectively, core in red, core border zone in orange and yellow, and contralateral side in green, Figure 3D) of uptake seen on the images. Interestingly, this difference in automatic segmentation patterns and the resulting ROIs for [^11^C]PK11195 and [^18^F]DPA-714 suggested differences in the pharmacokinetics of these ligands, as indeed using this segmentation method, ROIs of different shapes and volumes represent different pharmacokinetics by definition. As shown Figure 3D and Figure S1, the segmentation of the infarct core with [^18^F]DPA-714 appeared more consistent with the location of the MCAO territory and the expected infarct.

Replacing [^11^C]PK11195 in clinical imaging is challenging. Despite being highly lipophilic and displaying a poor signal to noise ratio, extensive work has been carried out in developing kinetic models for quantitative analysis [16]–[18], [45]–[47]. However, and although this is poorly reported in the literature but commonly accepted in the scientific community, easy and reliable production of [^11^C]PK11195 is known to be problematic, therefore ligands easier to radiolabel would also be welcome in the PET imaging field. Moreover, improved TSPO radioligands are still needed to visualise subtle changes in TSPO expression and there is still significant interest in developing new TSPO radiotracers from existing or new chemical classes [20], [48]–[50], despite the fact that several alternatives to [^11^C]PK11195 have been tested in preclinical animal models [19]. However, further validation of these tracers is required before clinical applications of these tracers would generalise. The need for clinical validation is exemplified by studies with [^11^C]PBR28, which demonstrated the presence of TSPO with different affinity in patients [22], [23] which, to our knowledge, is still undetected in animals. We do not have data on the ability of [^18^F]DPA-714 to discriminate low and high affinity binding sites observed in patients with other tracers. However, based the report looking at various TSPO ligands, including DPA-713 (4.4 fold difference in K_i_ between low and high binding sites) [23], it is likely that DPA-714 may also recognise different binding sites in human. Although the difference in affinity between the two binding sites is less for [^11^C]DPA-713 than for [^11^C]PBR28 (55 fold difference) or [^18^F]PBR06 (17 fold difference), the pharmacological properties of [^18^F]DPA-714 need to be assessed in that respect.

## Conclusions

Overall, our observations suggested that *i)* [^11^C]PK11195 and [^18^F]DPA-714 have different patterns of biodistribution/pharmacokinetics, which may be due to a different binding affinity to TSPO and *ii)* there might be advantages in using [^18^F]DPA-714 rather than [^11^C]PK11195 since we show here that the uptake in the contralateral healthy tissue (i.e. non-specific binding) is lower than for [^11^C]PK11195 hence leading to a better signal to noise ratio. Moreover, the longer half-life provided by [^18^F] labelling, allowing the tracer to be widely distributed from its production centre, also supports that [^18^F]DPA-714 is a suitable replacement for [^11^C]PK11195.

## Supporting Information

Figure S1 **Representative images of Claudin-5 and IgG immunohistochemistry (left panel) and [^11^C]PK11195 and [^18^F]DPA-74 PET images (right panel) co-registered with the MRI template of the same animal (rat #1) at similar coronal level.** Dotted and dashed lines represent the edge of the infarct/BBB disruption as detected by IgG diffusion in the brain parenchyma and lack of Claudin-5 immunostaining (tigh-junction disruption), overlap is seen for most of the area, although partial restoration of the tight-junction can be seen in the striatum (double-dotted/dashed line) with the Claudin-5 immunostaining (left middle panel). The approximate corresponding infarct delineation is also indicated on the PET images in the right panel.
(PDF)

Figure S2 **Representative [^11^C]PK11195 (left panel) and [^18^F]DPA-714 (right panel) PET summed images of 2 rats scanned with both tracers successively within 24 h, co-registered with the MRI template (A).** (**B**) T2 MRI template with simplified brain atlas ROI overlaid on the right hemisphere and corresponding coronal level of the Paxinos and Watson rat brain atlas (**C**). Arrow heads in panel A indicates area of heterogeneous tracer uptake in the caudate-putamen (dashed line on the PET images).
(PDF)

Table S1 **Comparison between [^11^C]PK11195 and [^18^F]DPA-714 R_1_ and binding potential (BP_ND_) for the core of the infarct in rats scanned with both [^11^C]PK11195 and [^18^F]DPA-714 and with visible infarct (n = 7).** * indicates significant differences between [^11^C]PK11195 and [^18^F]DPA-714 values, Wilcoxon test.
(DOCX)
