# Supplementary figures and images for: [18F]DPA-714: Direct Comparison with [11C]PK11195 in a Model of Cerebral Ischemia in Rats
Source: PLoS One. 2013 Feb 13;8(2):e56441. doi: 10.1371/journal.pone.0056441 (PMC3572061; doi:10.1371/journal.pone.0056441)

## Immunohistochemistry

Merged

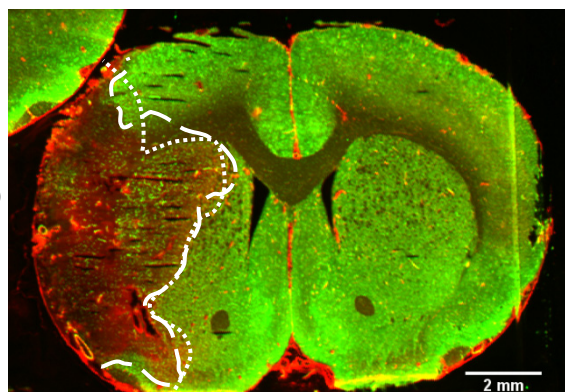

Claudin-5

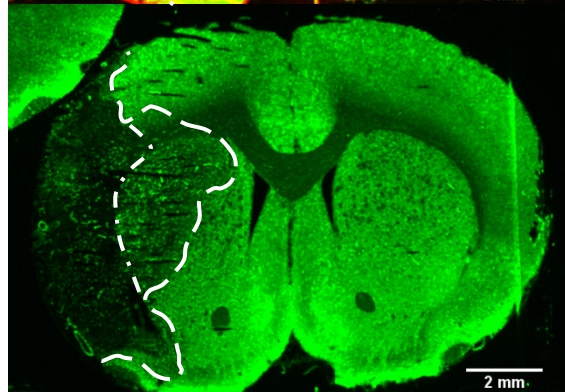

IgG

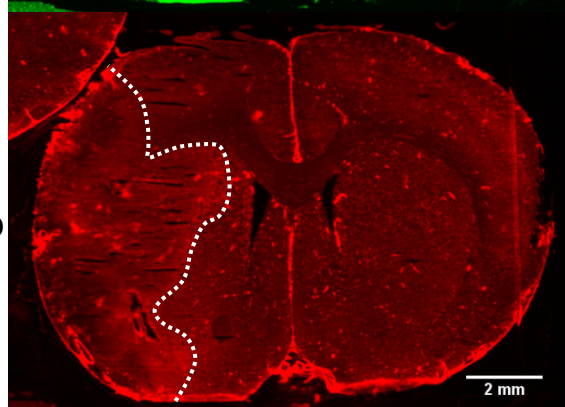

## PET imaging

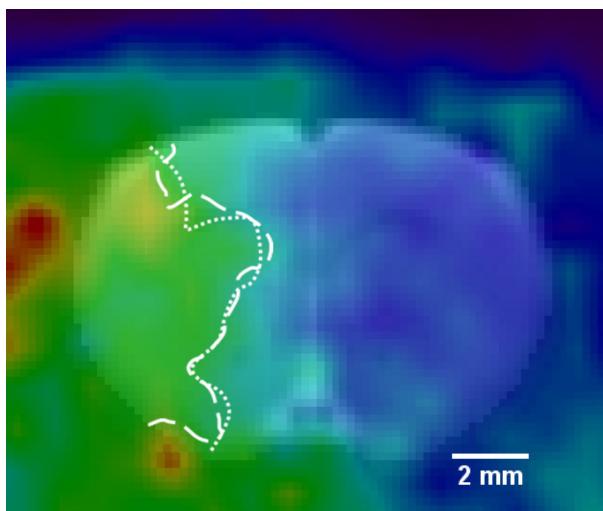

$[^{11}\text{C}]$ PK11195

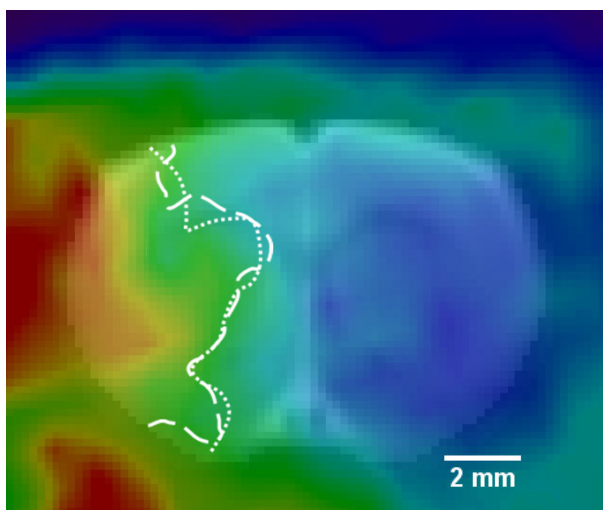

$[^{18}\text{F}]$ DPA-714

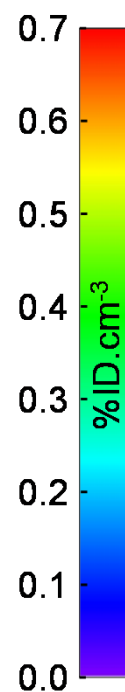

Supplement: Figure S1 — Representative images of Claudin-5 and IgG immunohistochemistry (left panel) and [11C]PK11195 and [18F]DPA-74 PET images (right panel) co-registered with the MRI template of the same animal (rat #1) at similar coronal level. Dotted and dashed lines represent the edge of the infarct/BBB disruption as detected by IgG diffusion in the brain parenchyma and lack of Claudin-5 immunostaining (tigh-junction disruption), overlap is seen for most of the area, although partial restoration of the tight-junction can be seen in the striatum (double-dotted/dashed line) with the Claudin-5 immunostaining (left middle panel). The approximate corresponding infarct delineation is also indicated on the PET images in the right panel. (PDF) [file pone.0056441.s001.pdf]

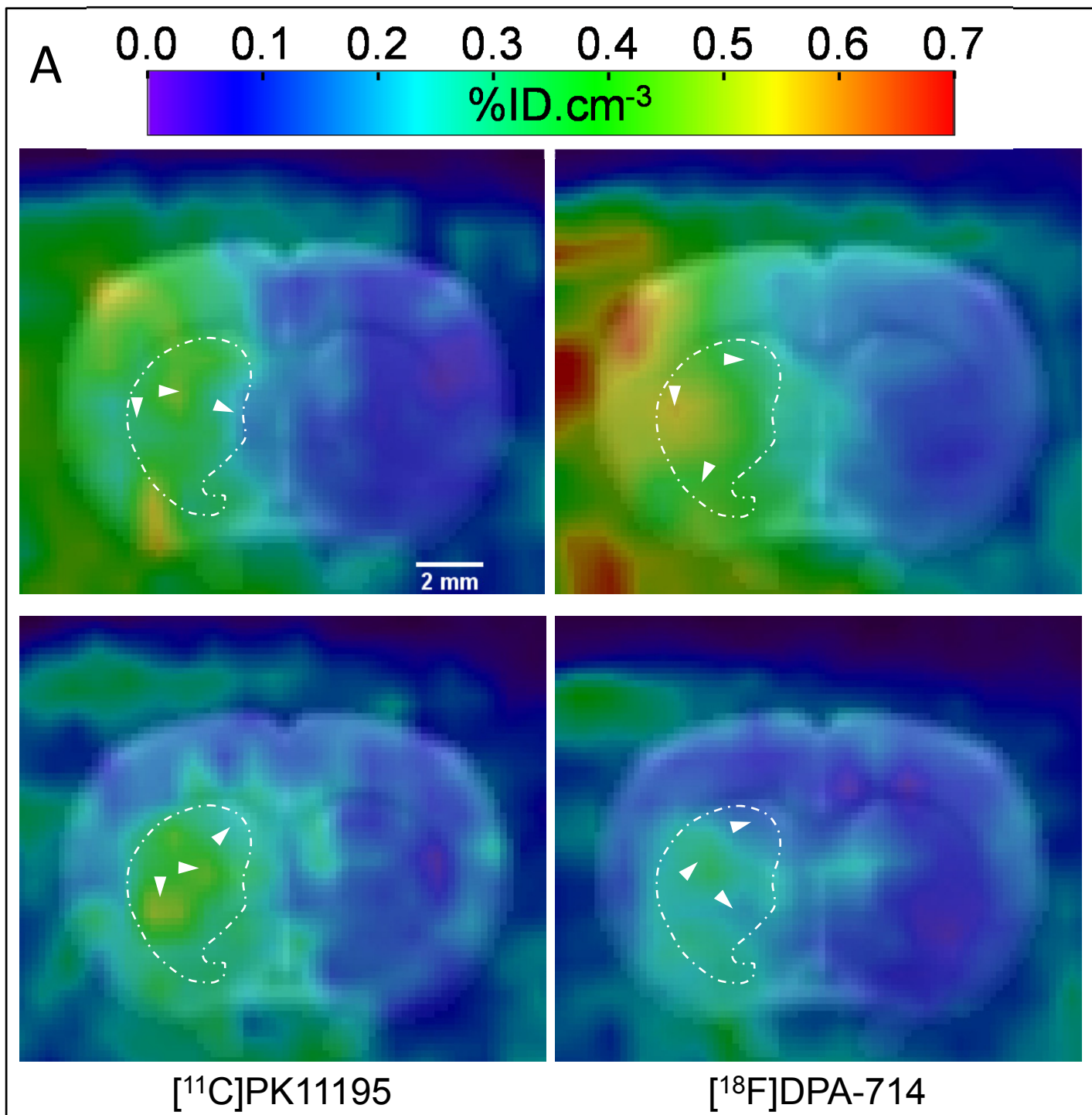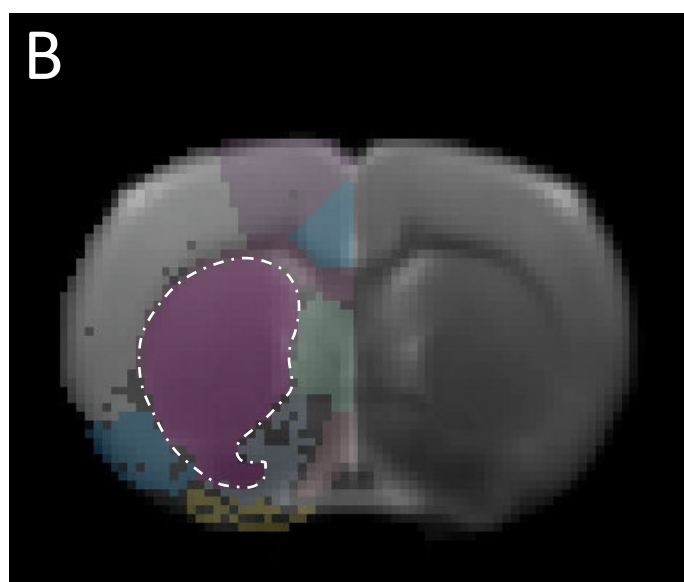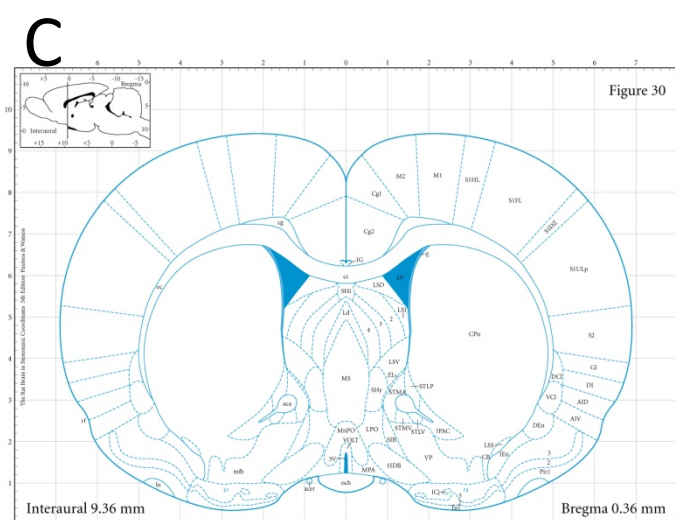

Supplement: Figure S2 — Representative [11C]PK11195 (left panel) and [18F]DPA-714 (right panel) PET summed images of 2 rats scanned with both tracers successively within 24 h, co-registered with the MRI template (A). (B) T2 MRI template with simplified brain atlas ROI overlaid on the right hemisphere and corresponding coronal level of the Paxinos and Watson rat brain atlas (C). Arrow heads in panel A indicates area of heterogeneous tracer uptake in the caudate-putamen (dashed line on the PET images). (PDF) [file pone.0056441.s002.pdf]
