# Supplementary material for: [18F]DPA-714: Direct Comparison with [11C]PK11195 in a Model of Cerebral Ischemia in Rats
Source: PLoS One. 2013 Feb 13;8(2):e56441. doi: 10.1371/journal.pone.0056441 (PMC3572061; doi:10.1371/journal.pone.0056441)
Supplement: Table S1 — Comparison between [11C]PK11195 and [18F]DPA-714 R1 and binding potential (BPND) for the core of the infarct in rats scanned with both [11C]PK11195 and [18F]DPA-714 and with visible infarct (n = 7). * indicates significant differences between [11C]PK11195 and [18F]DPA-714 values, Wilcoxon test. (DOCX) [file pone.0056441.s003.docx]

|  | | BP_ND_ core with | |
| --- | --- | --- | --- |
| ROIs | R_1_ | fitted R_1_ | R_1_ = 1 |
| [^11^C]PK11195 | 1.35±0.26 | 2.45±1.29 | 2.02±0.96 |
| [^18^F]DPA-714 | 1.75±0.73* | 4.43±3.69* | 3.07±1.98* |
